# The Role of the *Trypanosoma cruzi* TcNRBD1 Protein in Translation

**DOI:** 10.1371/journal.pone.0164650

**Published:** 2016-10-19

**Authors:** Camila Oliveira, Paulo Costa Carvalho, Lysangela Ronalte Alves, Samuel Goldenberg

**Affiliations:** Instituto Carlos Chagas, Fiocruz-Paraná, Rua Professor Algacyr Munhoz Mader, 3775, Cidade Industrial de Curitiba–CIC, 81350–010, Curitiba, Brasil; University of Toronto, CANADA

## Abstract

The regulation of gene expression in trypanosomatids occurs mainly at the post-transcriptional level. Despite the importance of this type of control in *Trypanosoma cruzi*, few RNA binding proteins have been characterized. The RRM domain (RNA Recognition Motif) is one of the most abundant domains found in RNA-binding proteins in higher eukaryotes. Proteins containing the RRM domain are involved in the majority of post-transcriptional processes regulating gene expression. In this work, we aimed to characterize the protein TcNRBD1 from *T*. *cruzi*. TcNRBD1 is an RNA-binding protein that contains 2 RRM domains and is the ortholog of the P34 and P37 proteins from *Trypanosoma brucei*. The TcNRBD1 protein is expressed in all developmental stages of *T*. *cruzi*, and its localization pattern is concentrated at the perinuclear region. TcNRBD1 is associated with polysomes and with the 80S monosomes. Furthermore, sequencing of the mRNAs bound to TcNRBD1 allowed the identification of several transcripts that encode ribosomal proteins. Immunoprecipitation assays followed by mass spectrometry showed that the protein complexes with several ribosomal proteins from both the 40S and 60S subunits. In summary, the results indicate that TcNRBD1 is associated with different parts of the translation process, either by regulating mRNAs that encode ribosomal proteins or by acting in some step of ribosome assembly in *T*. *cruzi*.

## Introduction

Chagas disease affects millions of people in America. The causative agent is *Trypanosoma cruzi*, a flagellate protozoan parasite of the Kinetoplastida order. The life cycle of the parasite is very complex, with two different hosts and four distinct developmental forms [1]. The mechanisms that regulate the differentiation steps are not well understood [2]. Currently, there is no evidence for the regulation of transcription initiation for protein-coding genes in *T*. *cruzi*; thus, the regulation of gene expression relies on post-transcriptional processes [3, 4]. The mature mRNAs are derived from the processing of polycistronic transcripts, and the distinct mRNAs are not functionally related, being produced at relatively constant levels although the transcripts are present at different levels in the cytoplasm [5, 6].

RNA binding proteins (RBPs) play a key-role in the posttranscriptional mechanisms involved in the regulation of gene expression. The RRM (RNA Recognition Motif) domain is abundant in RNA-binding proteins. Proteins containing this domain have affinity for single stranded RNA, and approximately 90 amino acids are involved in RNA recognition and binding [7]. An *in silico* analysis predicted approximately 80 proteins with RRM domains in *T*. *cruzi*; however, few proteins with the RNA recognition domain have been characterized [8]. A protein with this domain, TcUBP-1, plays an important role in the regulation of gene expression during differentiation of the parasite [9]. The RNA binding protein TcRBP40 binds to AG regions in the 3’-UTRs of the target mRNAs. Microarray data suggest that this protein binds to mRNAs that encode transmembrane proteins and that its localization changes during the life cycle of the parasite. It is located in reservosomes in epimastigote forms, whereas it is dispersed in the cytoplasm in amastigotes and trypomastigotes, suggesting a regulatory function [10]. In the case of TcRBP19, the protein seems to act in the regulation of the life cycle of the parasite because its overexpression affects the cell cycle progression of *T*. *cruzi* [11].

In previous work, TcNRBD1 was found associated with the polysomal fraction of epimastigotes and nutritionally stressed epimastigotes [12], and it was selected for further characterization. Our results indicate that TcNRBD1 might play a role in different steps of the translation process with a function distinct from that of the orthologous p34 in *T*. *brucei*.

## Materials and Methods

### Parasites

*T*. *cruzi* Dm28c [13] was used for all assays, including *in vitro* metacyclogenesis. The parasite was maintained in LIT (Liver Infusion Tryptose) medium. Epimastigotes at the late logarithmic growth phase were centrifuged at 7,000 x g for 5 minutes at 10°C. After centrifugation, the cells were subjected to nutritional stress in TAU medium (Triatomine Artificial Urine) for 2 hours. After the nutritional stress, 1 x 10^9^ cells were seeded in culture bottles of 300 cm^2^ containing 200 ml of TAU3AAG medium [13]. The metacyclic trypomastigotes were obtained by incubating stressed epimastigotes with TAU3AAG medium at a concentration of 5 x 10^6^ cells/mL for 96 hours at 28°C.

### Phylogenetic and molecular evolutionary analyses

Phylogenetic and molecular evolutionary analyses were conducted as previously described with slight modifications [14]. Briefly, we used ClustalW, with the default configuration for sequence alignment, and the phylogenetic tree was constructed by the Maximum Parsimony method with the default configuration. The evolutionary distances are expressed as the number of amino-acid substitutions per site. Bootstrap percentages (for 1000 replicas) are shown above the branches (when > 50).

### Cloning, recombinant protein expression and production of polyclonal antisera

The coding region of TcNRBD1 (KX827416) was amplified by PCR, cloned into the entry vector pDONR™221 (Gateway ®, Invitrogen), and subcloned into the pDEST17 vector for the production of a recombinant protein with a histidine tag at the N-terminus. The *Escherichia coli* BL21(DE3)pLysS strain transformed with the plasmid was induced with 1 mM IPTG at 30°C for 16 hours, and the recombinant protein was purified using a Ni-NTA column (Qiagen®—Hilden, Germany) according to the manufacturer's specifications. The antiserum against the recombinant protein was produced in rabbits (Laboratório de Imunologia—Centro de Biotecnologia—UFRGS).

Forward primer:

5’GGGGACAAGTTTGTACAAAAAAGCAGGCTGGAAGGAGATAATGCCCGCCAAGTCTGCC3’

Reverse primer:

5’GGGGACCACTTTGTACAAGAAAGCTGGGTCCTTCGTGTGGTTCTTTCTCTTGTTG3’.

### Immunofluorescence

Parasites at different stages of differentiation were centrifuged at 5,000 x g for 2 minutes, washed twice in 1x PBS, resuspended at a density of 10^6^ cells, and fixed with 4% paraformaldehyde in 1x PBS for 10 minutes. The cells (10^6^ parasites) were added to Teflon delimited field slides pretreated with poly-L-lysine and incubated in a humid chamber for 10 minutes. The cells were washed twice with 1x PBS, and this process was repeated in all the following steps. The cells were permeabilized with 0.1% Triton X-100 for 2 minutes and blocked with 1% PBS/BSA for 16 hours at 4°C, and this solution was also used to dilute antibodies. The parasites were incubated with the primary antibody for one hour at room temperature and washed three times by immersion in 1 x PBS for 5 minutes. The secondary antibody was added, and the incubation and washing steps were repeated. The cell nucleus and the kinetoplast were stained for 10 minutes with DAPI (1 g/mL) diluted in blocking solution. After this step, the slides were washed five times in 1x PBS, and 10 μL of n-propyl gallate was added. The slides were sealed with coverslips and observed with a fluorescence microscope. The serum dilutions were as follows: anti-TcNRBD1 1:300, Alexa Fluor 488 conjugated anti-rabbit secondary (Invitrogen®—Carlsbad, Califórnia, EUA) 1:400, and DAPI (4',6-diamidino-2-phenylindole dihydrochloride) 1:1000.

### Sucrose gradient sedimentation

Exponentially growing epimastigotes and epimastigotes under nutritional stress were incubated with 100 μg/ml cycloheximide for 10 minutes at room temperature or with 2 mM puromycin for one hour at 28°C, depending on the experimental procedure. The cells were centrifuged at 7,000 x g for 10 minutes at 4°C and washed twice with TKM buffer (10 mM Tris-HCl, pH 7.4, 10 mM MgCl_2_, 300 mM KCl, 100 mM cycloheximide). The cells were resuspended in 900 μl of TKM supplemented with 10 μg/ml heparin, 10 mM E-64 and 1 mM PMSF. The suspension was transferred to a new tube containing 100 mL of lysis buffer (TKM buffer containing 10% NP-40 and 2 M sucrose) and homogenized. The lysate was then centrifuged at 16,000 x g for 5 minutes at 4°C, and the supernatant was added to the top of linear sucrose gradients (15–55%) prepared in TKM buffer containing inhibitors. The gradient was centrifuged at 192,000 x g for 2 hours at 4°C. After centrifugation, the sucrose gradient fractions were collected (each fraction was 500 μL) using ISCO equipment [15].

### Immunoprecipitation–Proteomics

Twenty microliters of anti-NRBD1 were incubated with 50 μL of anti-rabbit IgG magnetic beads (New England Biolabs) for 2 hours at room temperature with continuous orbital shaking. The preimmune serum was incubated with the beads in the same conditions as a control for the specificity of the immunoprecipitation reaction. The magnetic beads were blocked with 1% BSA in PBS for 30 minutes and then washed twice with PBS. The cytoplasmic extracts of *T*. *cruzi* obtained from 10^9^ parasites were washed twice with PBS and incubated with 1 ml of hypotonic lysis buffer (50 mM Tris-HCl, pH 7.5, 1 mM MgCl_2_, and 150 mM NaCl) for 10 minutes followed by cavitation at 2,000 PSI for 30 minutes. The cytoplasmic extract was obtained after centrifugation at 7,000 x g for 20 minutes at 4°C. The extract was incubated for 2 hours at 4°C on an orbital shaker with magnetic beads previously linked to antibodies.

The immunoprecipitated complexes were collected and washed three times with hypotonic lysis buffer. The protein bound to beads was eluted with 40 μL of glycine 2 M, pH 2.5, and after elution, the pH of the solution was adjusted to 7.5–8.0. Proteins were solubilized in denaturation buffer (6 M urea, 2 M thiourea in 10 mM HEPES (pH 8.0)). The proteins were reduced with DTT (1 μg of DTT per 50 μg of protein) for 30 minutes at room temperature. Thereafter, iodoacetamide (IAA) (10% final concentration) was added, and the mixture was incubated for 20 minutes at room temperature followed by dilution of the samples with 4 volumes of digestion buffer (2 mg/mL ammonium bicarbonate in water). The peptides were obtained by adding trypsin (1 μg) and incubating the mixture overnight at room temperature. The reaction was stopped by acidification to pH< 2.5 with 100% TFA (final concentration 0.5% TFA). The peptides were purified using C18-StageTips previously equilibrated in buffer A (acetonitrile 5%, formic acid 0.1%). The peptides were washed in buffer A and then eluted in buffer B (acetonitrile 80%, formic acid 0.1%). After drying, the samples were resuspended in the same solution and analyzed by mass spectrometry (LTQ XL/Orbitrap (Thermo, EUA)).

### Immunoprecipitation–Ribonomics

After being washed twice with PBS, 10^9^ epimastigotes were lysed with buffer IMP1 (100 mM KCl, 5 mM MgCl_2_, 10 mM HEPES, pH 7.0, 0.5% Nonidet P40) to obtain the cytoplasmic extract, which was incubated with 50 μL of magnetic beads (as described above). The RNAs were eluted with buffer from the RLT RNeasy® kit according to the protocol "Animal Cells I" with the additional step of treatment with DNase in the column. After elution with 30 μl of water, the concentrations were measured with a spectrophotometer. For sequencing, 100–500 ng of purified mRNA was sequenced with the SOLiD 3 platform according to the manufacturer's recommendations. The analyses of the sequences were performed based on the mapping of sequences obtained from the reference genome (*Trypanosoma cruzi* AAHK01 assembly NCBI database) using the program CLC BioWorkbench^TM^ version 7.5.2. The reads alignment was performed as follows: Additional upstream and downstream sequences of 100 bases; minimum number of reads, 10; maximum number of mismatches, 2; nonspecific match limit, -2; use of colorspace encoding. We selected possible targets of TcNRBD1-mRNP with the β binomial statistical test reliability level to be ≥ 2% (false discovery rate, FDR). Furthermore, we used the absolute change value (fold-change) of 4 times (compared to the preimmune serum as a control) and RPKM (reads per kilo base per million) as criteria for the selection of differentially expressed genes. For Gene Ontology term enrichment analysis for the classification of transcripts we used the Blast2GO software (version 3.3.5) [16].

## Results

### Characterization of TcNRBD1

The TcNRBD1 protein was previously described in an analysis *in silico* of kinetoplastida proteins displaying RRM domains [8]. This protein shows 73% identity with p34 [17, 18] from *T*. *brucei*, and based on the role of p34 in this organism the TcNRBD1 protein was assumed to be a nuclear RNA binding protein. In addition, the TcNRBD1 protein is closely related to p37 from *T*. *brucei*, another RNA-binding protein that was found to be essential for the ribosomal biogenesis and survival of the parasite. A protein named TcP37/NRBD has recently been described, and it shows 89% identity to TcNRBD1 except for a few amino acid differences in the N-termini of the proteins [19]. In that report, TcP37/NRBD was described as a homolog of TbP34 and TbP37. Using antibodies produced against the *T*. *brucei* P34/P37 proteins, the authors observed nuclear and nucleolar localizations for the protein and described a direct interaction with *T*. *cruzi* 5S rRNA but not with polyadenylated RNA.

**Fig 1:**
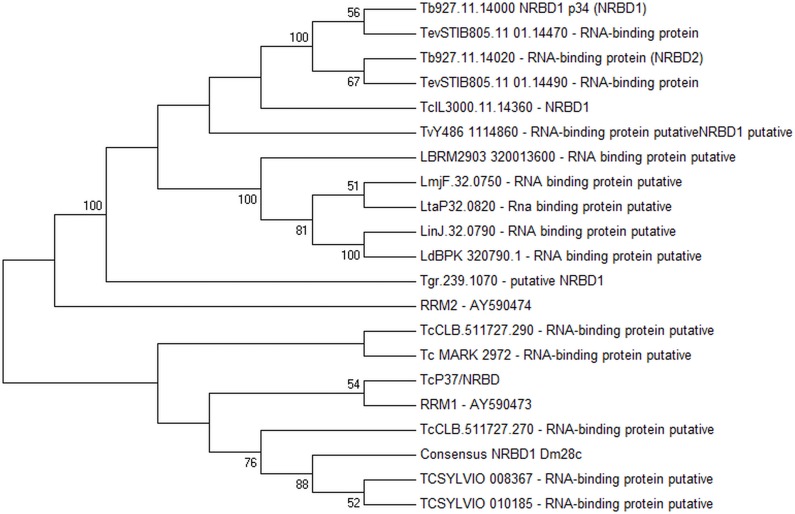
The evolutionary history was inferred using the Maximum Parsimony method. The bootstrap consensus tree inferred from 1000 replicates is taken to represent the evolutionary history of the taxa analyzed. Branches corresponding to partitions reproduced in less than 50% bootstrap replicates are collapsed. The percentage of replicate trees in which the associated taxa clustered together in the bootstrap test (1000 replicates) are shown next to the branches. The MP tree was obtained using the Subtree-Pruning-Regrafting (SPR) algorithm with search level 1 in which the initial trees were obtained by the random addition of sequences (10 replicates). The analysis involved 21 amino acid sequences. There were a total of 289 positions in the final dataset. Evolutionary analyses were conducted in MEGA7 [34].

The genome sequence data available for *T*.*cruzi* Dm28c has low coverage and some regions have not been sequenced, as is the case of TcNRBD1. Hence we grouped the RNA-seq data available from our group (transcriptomics and ribonomics) and aligned against CL Brener genome, selecting the region containing the genes TcCLB.511727.260 through TcCLB.511727.300 as reference. We performed a directed de novo assembly using only the reads that mapped on the reference and were able to identify the TcNRBD1 gene sequence (accession number KX827416). To compare and infer the relation between these two proteins (TcP37/NRBD and TcNRBD1), we performed a phylogenetic analysis of their encoding genes (Fig 1 and S1 and S2 Figs). It is possible to observe that the TcNRBD1 (consensus NRBD1 Dm28C) is more closely related to the NRBD1 gene from Sylvio strain than to the one from CL Brener strain or the *T*. *brucei* P34 (NRBD1) and P37 (NRBD2) and the maximum parsimony corroborated our results that they are different genes. In addition, the results demonstrated that TcP37/NRBD and TcNRBD1 are also distinct genes. Despite their high identity that could have arisen from gene duplication, these proteins might exert distinct functions in *T*. *cruzi*. In the case of NRBD1 there are at least 2 copies of the gene in the *T*. *cruzi* CL Brener genome, and the genes encoding these two proteins are organized in tandem.

**Fig 2:**
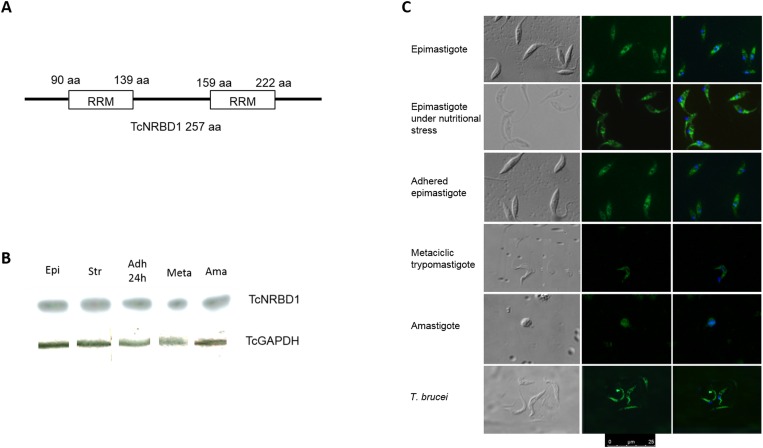
(A) Scheme of the TcNRBD1 gene. (B) TcNRBD1 expression throughout the parasite's life cycle: 1 –epimastigote in exponential growth, 2—epimastigote under nutritional stress, 3 –epimastigotes adhered to the substrate for 24 hours, 4—metacyclic trypomastigote, 5—amastigote (5x10^6^ parasites in each lane). Secondary antibodies: anti-rabbit peroxidase (1:1000) and anti-mouse phosphatase (1:10000). (C) Immunolocalization of TcNRBD1 during the *T*. *cruzi* lifecycle. The bottom panel is the promastigote form of *T*. *brucei*. The primary antibody was diluted 1:300. The kinetoplast and nucleus were stained with DAPI (4',6-diamidino-2-phenylindole dihydrochloride) 1:1000. The secondary antibody was Alexa Fluor 488 conjugated anti-rabbit diluted 1:400. Field 1, DIC; Field 2, immunofluorescence of TcNRBD1; Field 3, DAPI.

TcNRBD1 is a 28 kDa protein and has 2 RRM domains in its structure spanning 90–139 aa and 159–222 aa (Fig 2A). The protein is expressed throughout the parasite`s life cycle (Fig 2B), and the localization of TcNRBD1 showed a concentrated pattern in the perinuclear region and a granular pattern in the cytoplasm in all the developmental stages analyzed (Fig 2C). Immunolocalization using the anti-TcNRBD1 was also performed with the *T*. *brucei* 427 strain (Fig 2C) to determine whether P34 and P37 are orthologs of TcNRBD1 in *T*. *brucei* and where they localize. A cytoplasmic localization was observed in both *T*. *cruzi* and *T*. *brucei* (Fig 2C). In addition, the TcNRBD1 anti-serum recognized only one protein in the *T*. *brucei* protein extract (S3 Fig). We then decided to extend the permeabilization time to assess whether the observed localization in *T*. *cruzi* was due to a technical reason. Even with the permeabilization time extended from 1 to 5 minutes, we did not observe any alteration in the protein localization pattern (S4 Fig). In addition, we performed a colocalization assay of TcNRBD1 with TcNUP-1 [20], a known protein of the nuclear pore. The results indicated that TcNRBD1 is localized in the cytoplasm and in the nuclear vicinity but not inside the nucleus (S5 Fig). We also observed that in CL Brener strain the TcNRBD1 protein had the same localization as Dm28c strain (S5 Fig).

### TcNRBD1 is associated with polysomes

**Fig 3:**
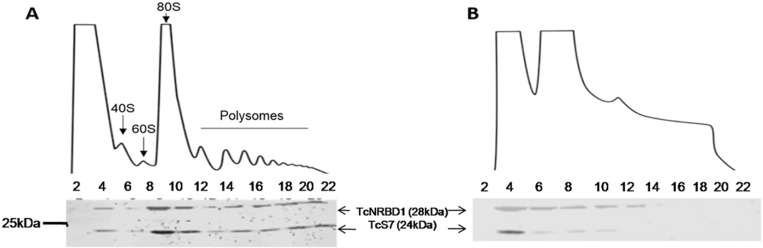
Western blot of the sucrose gradient fractions: (A) epimastigotes treated with cycloheximide, (B) epimastigotes treated with puromycin. The positive controls used were anti-P0 (1:500) and anti-S7 (1:500). Only the odd fractions (1–21) collected from the gradient were loaded on the gel.

Previous results indicated that TcNRBD1 was associated with polysomal fractions [12]. To confirm that observation and to determine the behavior of TcNRBD1 in the course of the nutritional stress that precedes *T*. *cruzi* differentiation, we investigated the migration pattern of TcNRBD1 in sucrose gradients. The results showed that TcNRBD1 was identified in the light to the heavy fractions of the gradient in the presence of cycloheximide, which preserves the polysomes by inhibiting their run-off (Fig 3A). However, upon dissociation of the polysomes with puromycin, we observed a shift in the sucrose gradient migration pattern of TcNRBD1, which was identified only in the lighter fractions with the ribosomal proteins P0 and S7 used as controls (Fig 3B).

### Identification of the proteins associated with TcNRBD1

Because TcNRBD1 is associated with the polysomes, we next investigated the proteins associated with TcNRBD1 by immunoprecipitation followed by mass spectrometry analysis, using epimastigotes and epimastigotes under nutritional stress. The preimmune serum was used in parallel as a control under the same conditions. The results were compared using the program PatternLab for Proteomics [21, 22]. Confirming our previous results, most of the proteins identified were ribosomal proteins in both unstressed and stressed epimastigotes (S1 and S2 Tables). For stressed epimastigotes, more than half of the proteins identified were ribosomal proteins from both the small (40S) and the large (60S) subunits.

### Identification of the mRNAs associated with the TcNRBD1-mRNP

**Fig 4:**
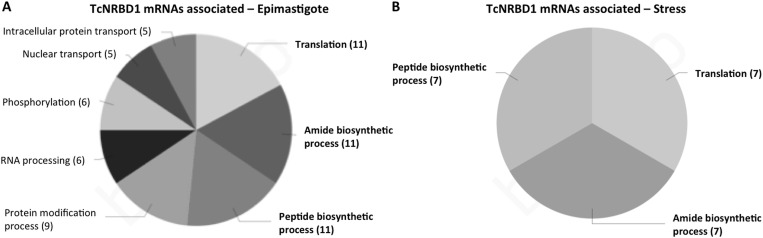
Gene Ontology classification of unstressed (A) and stressed epimastigotes (B) transcripts associated with TcNRBD1. The pie chart refers to level 6 in Biological Process. In bold the terms common to both conditions.

The RNAs associated with TcNRBD1 were also identified by ribonomic analysis of ribonucleoprotein complexes immunoprecipitated with the antiserum against TcNRBD1. As a negative control, we used the preimmune serum. Using an RPKM value of at least 100, we identified 142 transcripts as more abundant, by a fold-change of at least four, in the TcNRBD1-mRNP from epimastigotes compared to the control (FDR >0.02). For stressed epimastigotes, 54 mRNAs were identified as being more abundant, by a fold change of at least four, in the TcNRBD1-mRNP than in the control. In both cases, the most abundant transcripts contained code for ribosomal proteins, corroborating the assumption that TcNRBD1 is linked to the ribosomal complex (Fig 4). The results with all the mRNAs identified are listed in the S3 and S4 Tables. We validated the RNA-seq by immunoprecipitation followed by RT-PCR (S6 Fig). The sequencing data are available at NCBI Sequence read archive under the accession SRS404036.

## Discussion

The *T*. *cruzi* TcNRBD1 protein shows 73% identity to the related proteins p34 and p37 (Fig 1). These proteins showed a dispersed pattern in the cytoplasm, although a nuclear localization has been described in *T*. *brucei* [18], and it was proposed that they might play a role in ribosome biogenesis. Co-immunoprecipitation and sucrose gradient sedimentation profiles showed that the p34/p37 proteins interact with 5S rRNA [23]. A knockdown of p34/p37 by RNAi resulted in a dramatic decrease of the 5S rRNA levels as well as an accumulation of the large ribosomal subunit [23]. Despite the high identity between the proteins from *T*. *cruzi* and *T*. *brucei*, we observed that TcNRBD1 in *T*. *cruzi* showed an exclusive cytoplasmic localization. Furthermore, *T*. *brucei* displays only one copy of the p34 and p37 genes in the genome, whereas *T*. *cruzi* has at least two identical copies of TcNRBD1 in CL Brener.

Sucrose gradient sedimentation showed that the protein TcNRBD1 migrated to the fraction corresponding to the 80S complex; this pattern of migration is characteristic of ribosomal proteins or proteins associated with the translation machinery [24]. One example of this migration pattern is the RACK1 protein. In yeast [25], plants [26] and mammalian cells [27], RACK1 is one of the main components of ribosomes, and in *T*. *brucei* it is associated with the translation elongation factor 1A (eEF1-alpha) [28].

Proteomic analysis showed that a large number of ribosomal proteins were identified associated with TcNRBD1 both in epimastigotes and in epimastigotes under nutritional stress. Accordingly, in epimastigotes under stress, approximately 70% of the identified proteins were considered to be ribosomal from either the small (40S) or the large subunits (60S). These results reinforce the hypothesis of the association of TcNRBD1 with the translation machinery, where it might be acting in ribosome assembly and in other translation steps. RNA-binding proteins can play roles in different steps of translation regulation, including ribosome biogenesis, rRNA processing and initiation, elongation and termination of translation [29–31]. The RRM domain in the PRT1 protein, a member of the translation initiation complex, together with the eIF3 protein and TIF32 factors, is associated with the 40S subunit of the ribosome, modulating and stabilizing the translation complex. To demonstrate this association, removal of the RRM domain resulted in the dissociation of the whole complex, thus preventing the association of the 40S subunit and the formation of the ribosome [32]. Hence, TcNRBD1 might be acting in ribosome assembly and in other translation steps.

The analysis of the mRNAs bound to TcNRBD1-mRNP allowed the identification of several transcripts that encode ribosomal (Fig 4A and 4B). It is tempting to speculate that TcNRBD1 might be regulating the expression level of some ribosomal proteins that would ultimately modulate translation. In addition, TcNRBD1 might play a role in the control of general translation because we observed the association of ribosomal proteins and translation initiation factors with TcNRBD1. Some RNA binding proteins, including the Alba2 and Alba3 proteins from *T*. *brucei*, also act in the formation of the translation initiation complex and participate in the regulation of translation [33]. Pull down assays demonstrated the association of Alba2 with PABP and the ribosomal protein P0, whereas Alba3 interacts with cap binding protein eIF4E4. These proteins are also recruited to stress granules when cells are subjected to nutritional stress [33].

Despite the similarities of *T*. *brucei* p34/p37 and *T*. *cruzi* P37/NRBD1 to TcNRBD1, we found that, whereas these proteins have a role in ribosomal biogenesis in the nuclei [23], TcNRBD1 has a different role. The protein is associated with ribosomal proteins as well as with some mRNAs encoding ribosomal proteins in the cytoplasm. This gene is present in 2 copies in *T*. *cruzi*, suggesting a possible genic duplication. In summary, our results suggest an involvement of TcNRBD1 in the regulation of gene expression in *T*. *cruzi* by associating with the translation machinery.

## Supporting Information

S1 Fig Clustal W alignment showing the similarities and differences between the proteins from the organisms used in the phylogenetic analysis in the Fig 1. (TIF)

S2 Fig Pairwise comparison with a gradient matrix. Upper comparison gradient is related to gaps (the number of alignment positions where one sequence has a gap and the other does not). Lower comparison gradient is related to differences (the number of alignment positions where the two sequences agree). The blue color means the minimum values and the red color means the maximum values.
(TIF)

S3 Fig Western blot analysis of promastigote form of *T*. *brucei* 427 strain protein extract revealed with anti-TcNRBD1. 5x10^6^ parasites in the lane, The TcNRBD1 antibody was diluted 1:300 and the secondary antibody anti-rabbit phosphatase was diluted 1:10000. The molecular weight are indicated in kDa.
(TIF)

S4 Fig Immunolocalization of TcNRBD1 in *T*. *cruzi* (strain Dm28c) epimastigote. The primary antibody was diluted 1:300. The kinetoplast and nucleus were stained with DAPI (4',6-diamidino-2-phenylindole dihydrochloride) 1:1000. The secondary antibody was Alexa Fluor 488 conjugated anti-rabbit diluted 1:400. Field 1, DIC; Field 2, immunofluorescence of TcNRBD1; Field 3, DAPI. The parasites were permeabilized for different times (3 or 5 minutes).
(TIF)

S5 Fig Colocalization of TcNRBD1 and TcNUP1 (Dm28c) epimastigote (top panel). The anti-NRBD1 was diluted 1:300 and the anti-NUP1 was diluted 1:500. Immunolocalization of TcNRBD1 in *T*. *cruzi* (CL Brener) epimastigote (bottom panel). The kinetoplast and nucleus were stained with DAPI (4',6-diamidino-2-phenylindole dihydrochloride) 1:1000. The secondary antibodies were Alexa 488 rabbit and Alexa 594 mouse 1:400 anti-fluoride. Field 1, the immunofluorescence of TcNRBD1 and TcNUP1; Field 2, DAPI; Field 3, DIC.
(TIF)

S6 Fig Reverse transcriptase PCR after TcNRBD1 immunoprecipitation. (A) 1 –TcCLB.511211.160: heat shock protein 70 (hsp70), putative (2217bp); 2 –TcCLB.510441.30: trans-sialidase (pseudogene), putative; 3 –TcCLB.511753.120: L-threonine 4-dehydrogenase, putative (999bp); 5 –TcCLB.507681.160: 40S ribosomal protein S24E (414bp); 6 –TcCLB.409479.10: ribosomal RNA small subunit (298bp). (B) Immunoprecipitation controls. 1 preimmune sérum immunoprecipitation tested with TcCLB.409479.10: ribosomal RNA small subunit; 2 –RT-PCR without the RNA template; 3 –RT-PCR performed without the Super script III enzyme. Size in base pairs.
(TIF)

S1 Table Proteins identified by proteomic analysis in epimastigotes. (PDF)

S2 Table Proteins identified by proteomic analysis in epimastigotes under nutritional stress. (PDF)

S3 Table mRNAs associated to TcNRBD1-mRNP in epimastigotes. (PDF)

S4 Table mRNAs associated to TcNRBD1-mRNP in epimastigotes under nutritional stress. (PDF)
