# Supplementary figures and images for: The Role of the Trypanosoma cruzi TcNRBD1 Protein in Translation
Source: PLoS One. 2016 Oct 19;11(10):e0164650. doi: 10.1371/journal.pone.0164650 (PMC5070865; doi:10.1371/journal.pone.0164650)

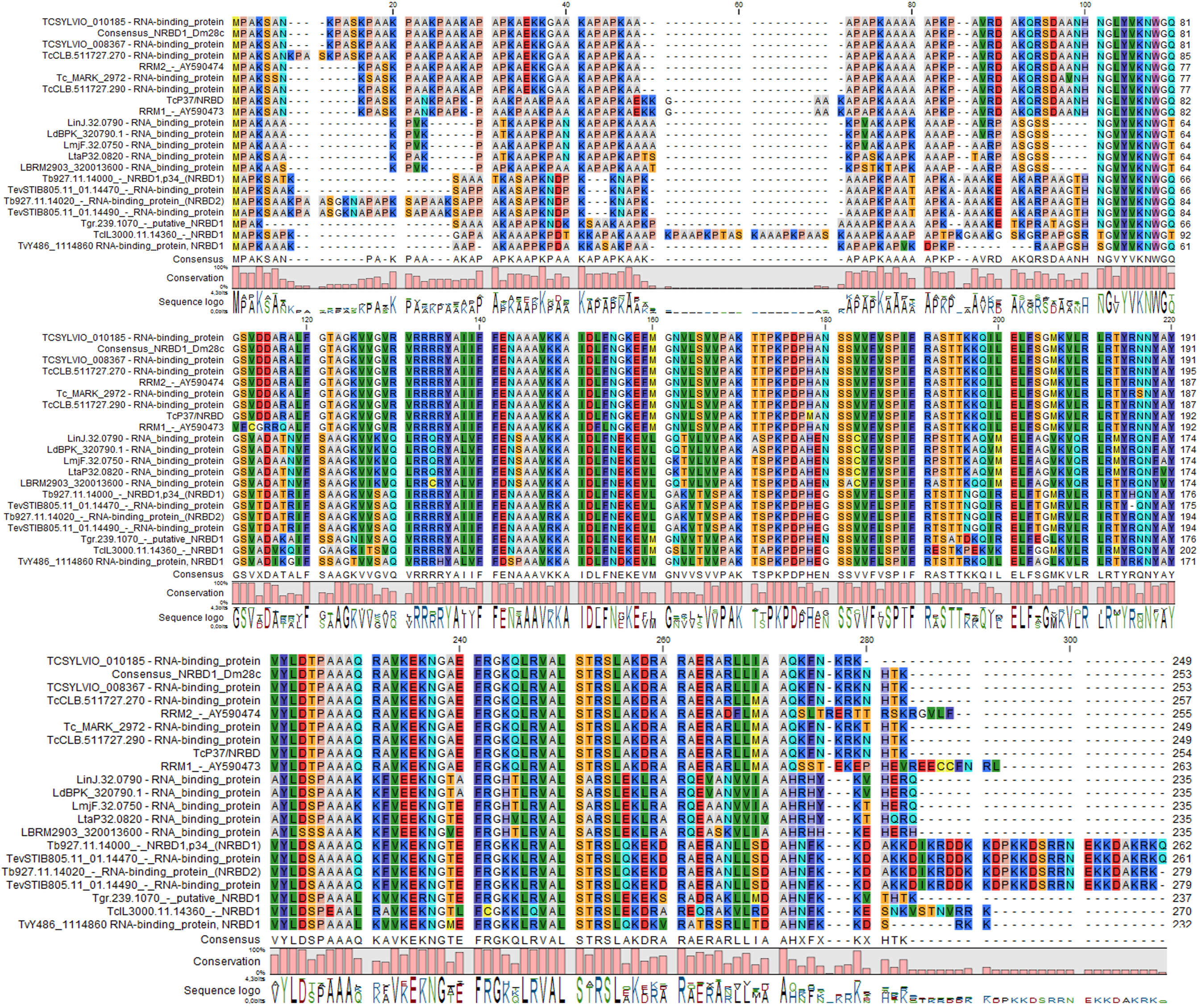

Supplement: S1 Fig — (TIF) [file pone.0164650.s001.tif]

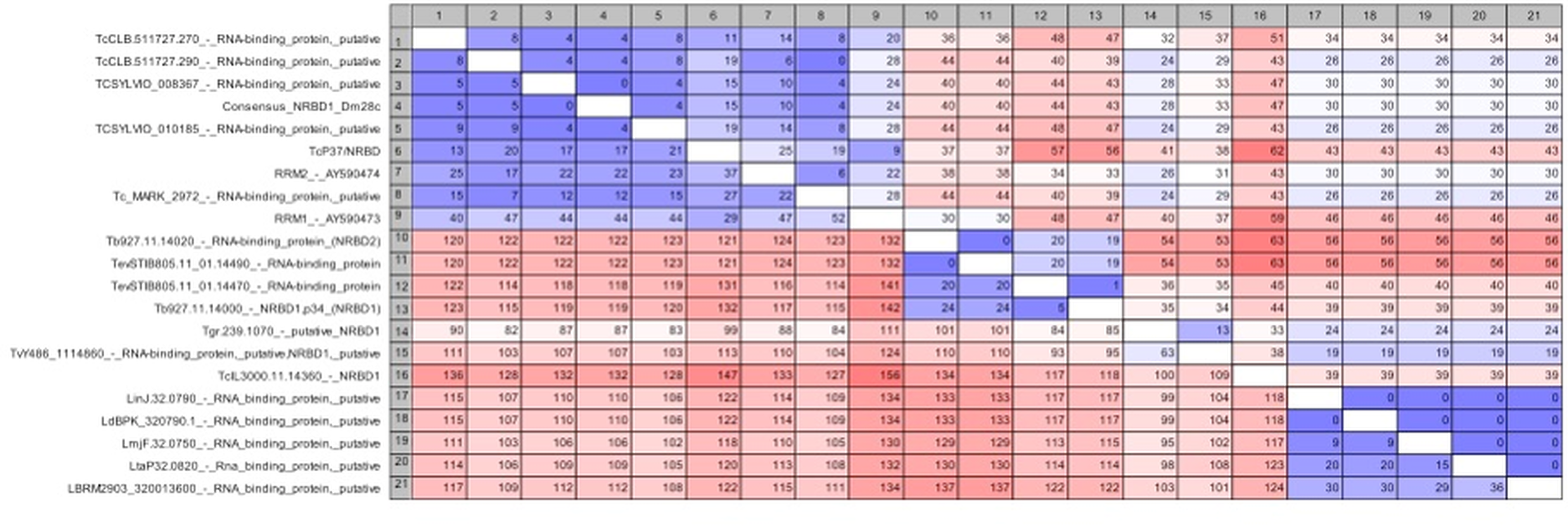

Supplement: S2 Fig — Upper comparison gradient is related to gaps (the number of alignment positions where one sequence has a gap and the other does not). Lower comparison gradient is related to differences (the number of alignment positions where the two sequences agree). The blue color means the minimum values and the red color means the maximum values. (TIF) [file pone.0164650.s002.tif]

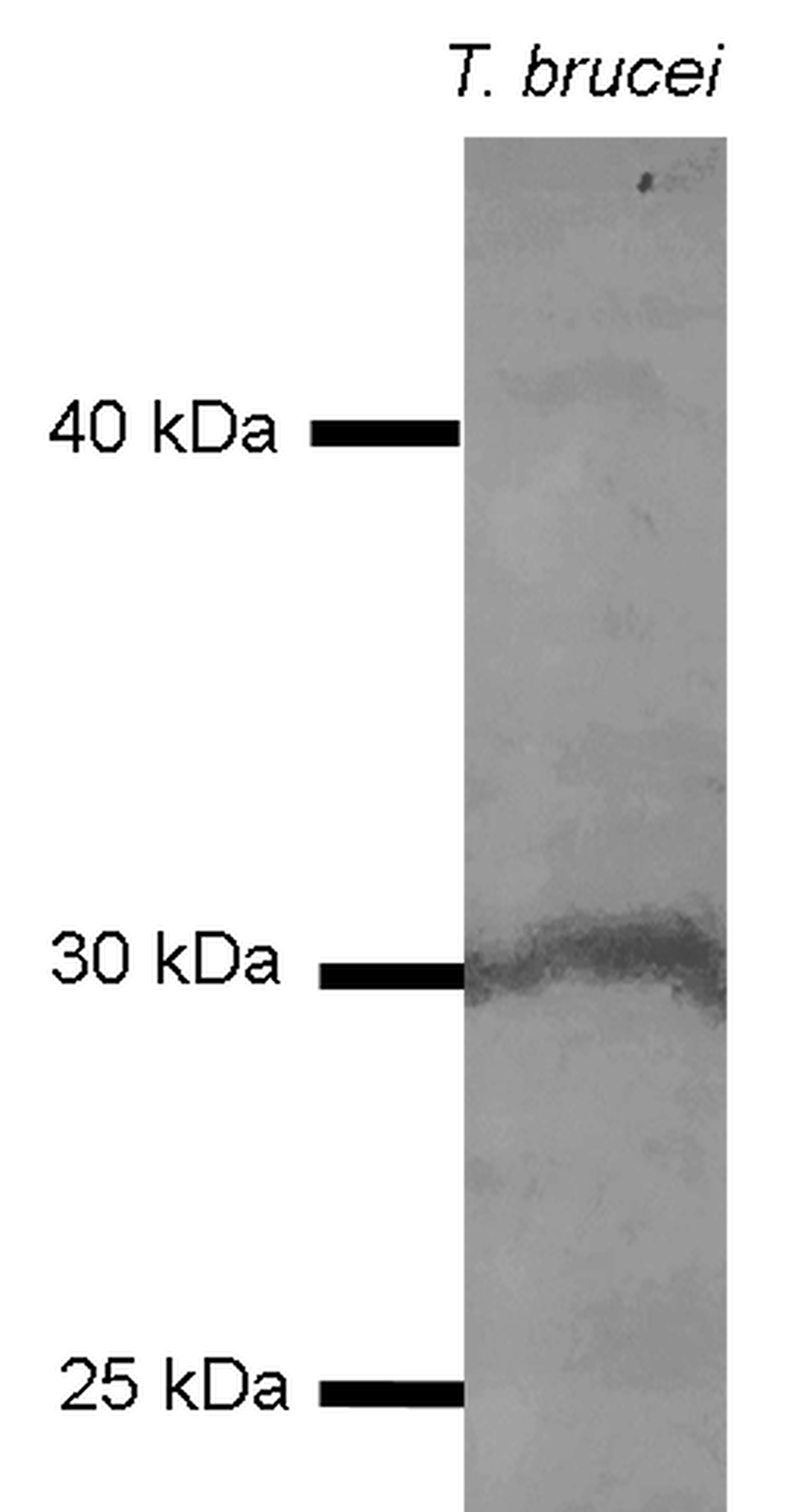

Supplement: S3 Fig — 5x10^6^ parasites in the lane, The TcNRBD1 antibody was diluted 1:300 and the secondary antibody anti-rabbit phosphatase was diluted 1:10000. The molecular weight are indicated in kDa. (TIF) [file pone.0164650.s003.tif]

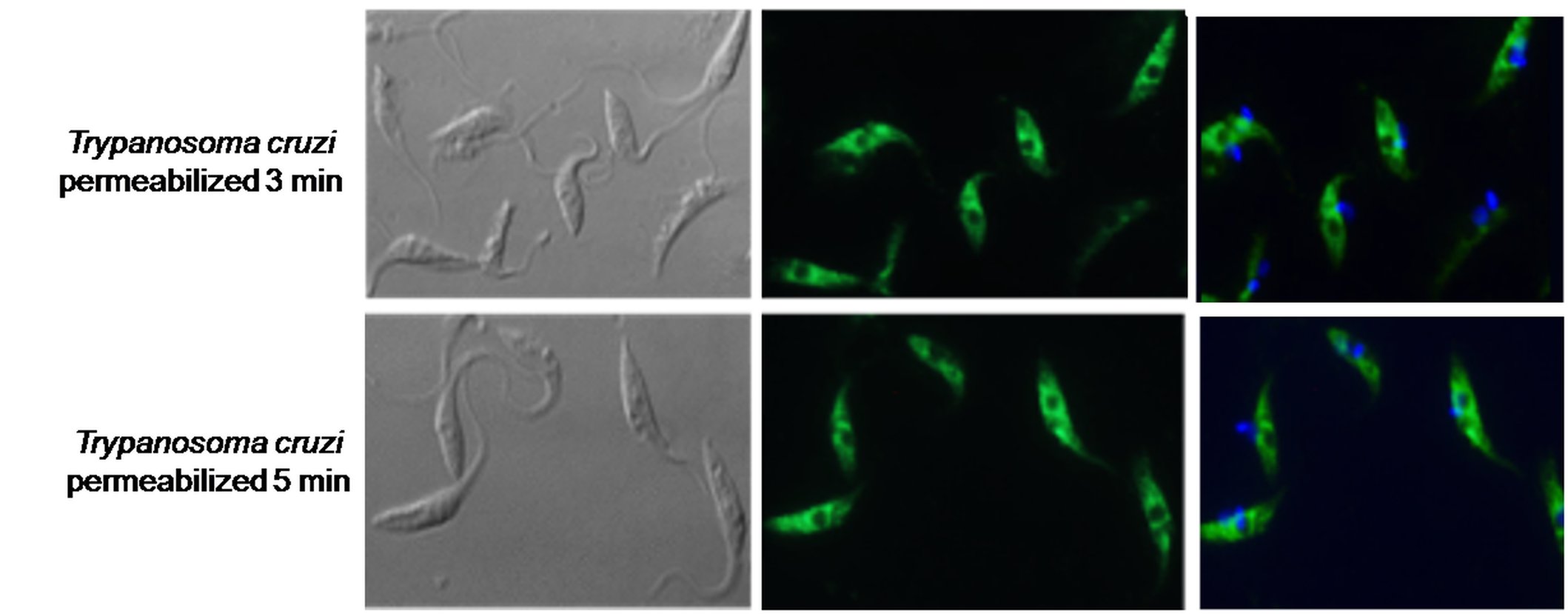

Supplement: S4 Fig — The primary antibody was diluted 1:300. The kinetoplast and nucleus were stained with DAPI (4',6-diamidino-2-phenylindole dihydrochloride) 1:1000. The secondary antibody was Alexa Fluor 488 conjugated anti-rabbit diluted 1:400. Field 1, DIC; Field 2, immunofluorescence of TcNRBD1; Field 3, DAPI. The parasites were permeabilized for different times (3 or 5 minutes). (TIF) [file pone.0164650.s004.tif]

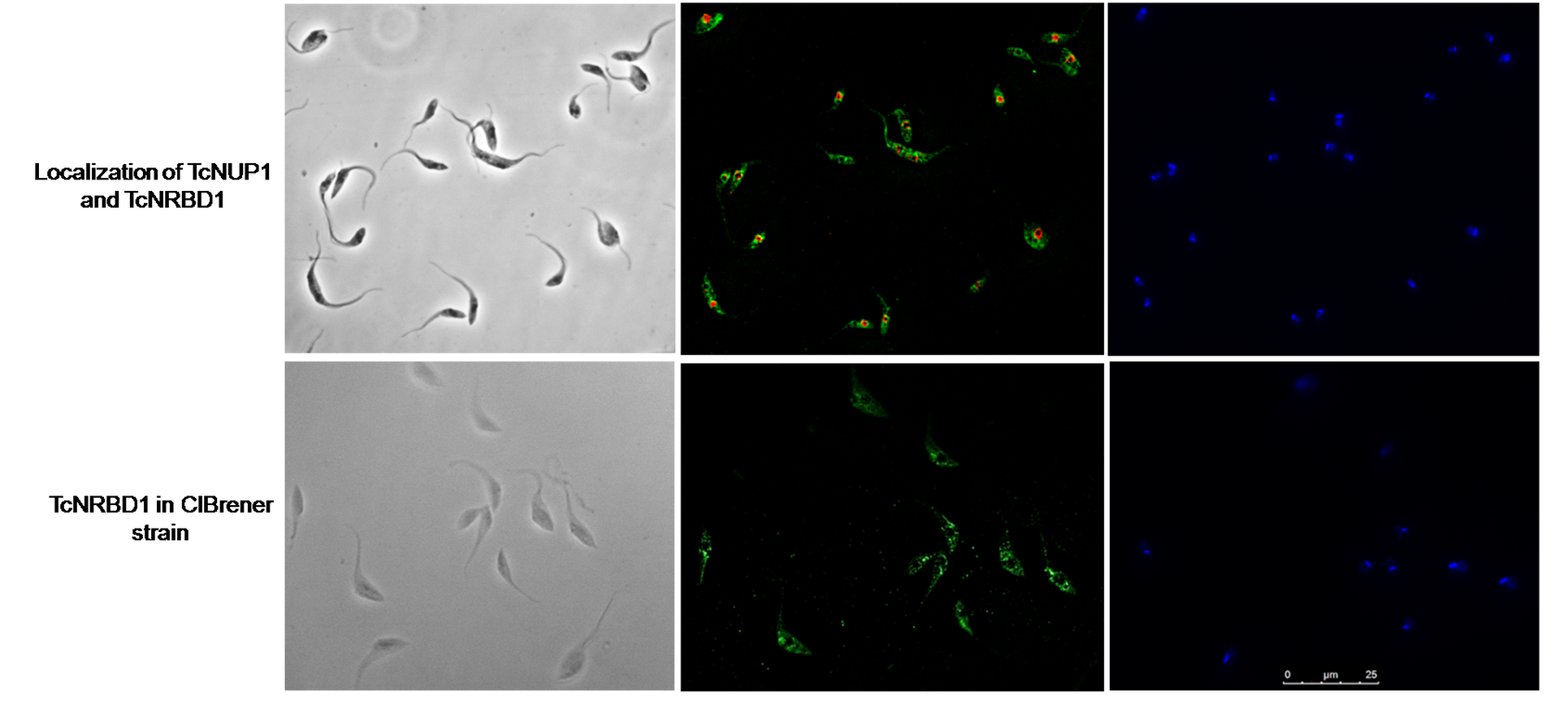

Supplement: S5 Fig — The anti-NRBD1 was diluted 1:300 and the anti-NUP1 was diluted 1:500. Immunolocalization of TcNRBD1 in T. cruzi (CL Brener) epimastigote (bottom panel). The kinetoplast and nucleus were stained with DAPI (4',6-diamidino-2-phenylindole dihydrochloride) 1:1000. The secondary antibodies were Alexa 488 rabbit and Alexa 594 mouse 1:400 anti-fluoride. Field 1, the immunofluorescence of TcNRBD1 and TcNUP1; Field 2, DAPI; Field 3, DIC. (TIF) [file pone.0164650.s005.tif]

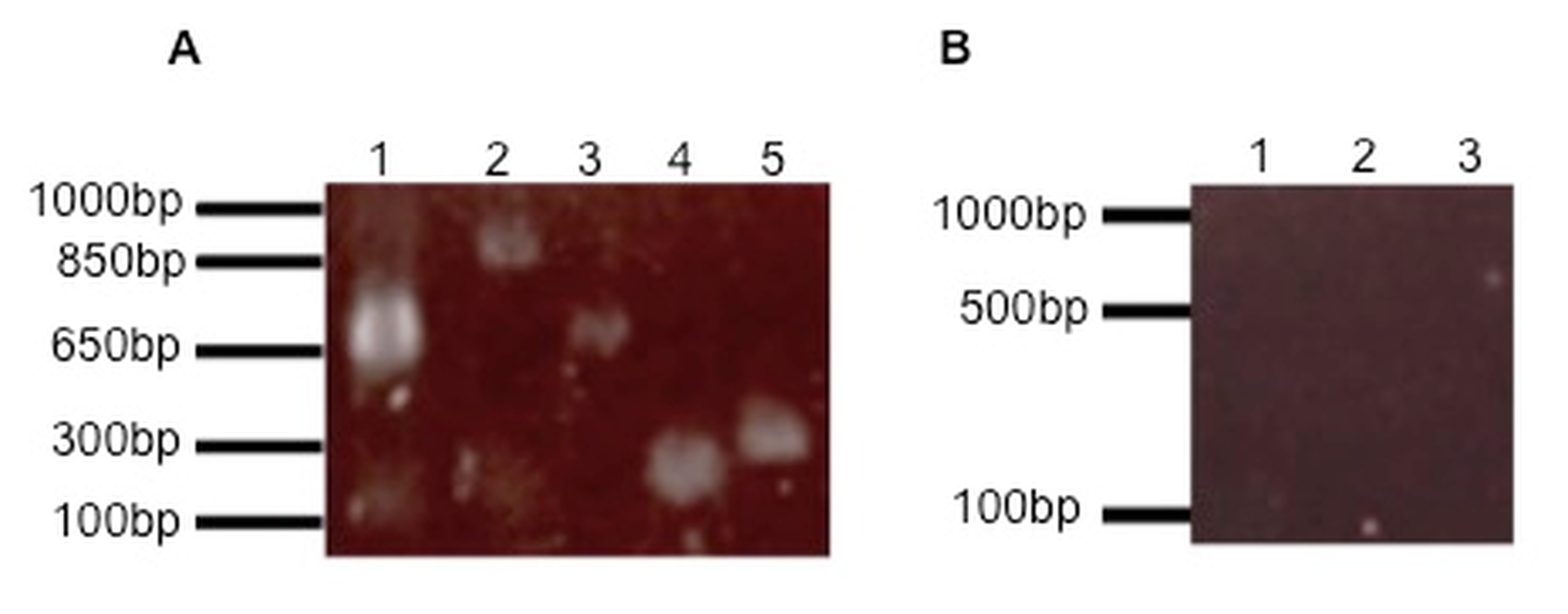

Supplement: S6 Fig — (A) 1 –TcCLB.511211.160: heat shock protein 70 (hsp70), putative (2217bp); 2 –TcCLB.510441.30: trans-sialidase (pseudogene), putative; 3 –TcCLB.511753.120: L-threonine 4-dehydrogenase, putative (999bp); 5 –TcCLB.507681.160: 40S ribosomal protein S24E (414bp); 6 –TcCLB.409479.10: ribosomal RNA small subunit (298bp). (B) Immunoprecipitation controls. 1 preimmune sérum immunoprecipitation tested with TcCLB.409479.10: ribosomal RNA small subunit; 2 –RT-PCR without the RNA template; 3 –RT-PCR performed without the Super script III enzyme. Size in base pairs. (TIF) [file pone.0164650.s006.tif]
