# Supplementary material for: The Role of the Trypanosoma cruzi TcNRBD1 Protein in Translation
Source: PLoS One. 2016 Oct 19;11(10):e0164650. doi: 10.1371/journal.pone.0164650 (PMC5070865; doi:10.1371/journal.pone.0164650)
Supplement: S1 Table — (PDF) [file pone.0164650.s007.pdf]

**S1 Table:** Proteins identified by proteomic analysis in epimastigotes.

| ID | Description | Spectral count |
|---|---|---|
| Tc00.1047053510593.10 | Hypothetical protein, conserved | 7 |
| Tc00.1047053511211.40 | Molybdenium cofactor biosynthesis, putative | 6 |
| Tc00.1047053509717.20<br>Tc00.1047053510105.170 | Dihydrolipoamide acetyltransferase precursor, putative | 9 |
| Tc00.1047053510565.30<br>Tc00.1047053508503.20 | Cytochrome c oxidase subunit V, putative | 11 |
| Tc00.1047053506943.60<br>Tc00.1047053509065.70<br>Tc00.1047053506943.50<br>Tc00.1047053509065.60 | Glyceraldehyde 3-phophate dehydrogenase, putative | 7 |
| Tc00.1047053510877.40<br>Tc00.1047053510877.30 | Hypothetical protein, conserved | 18 |
| Tc00.1047053511231.80<br>Tc00.1047053504017.10 | Basic transcription factor 3a, putative | 10 |
| Tc00.1047053511319.20<br>Tc00.1047053510055.50 | Hypothetical protein, conserved | 6 |
| Tc00.1047053503675.20<br>Tc00.1047053506337.30 | SNF7-like protein, putative | 9 |
| Tc00.1047053511727.270<br>Tc00.1047053511727.290 | RNA-binding protein, putative | 9 |
| Tc00.1047055310029.70<br>Tc00.1047053510973.30<br>Tc00.1047053511189.30<br>Tc00.1047053511291.90 | 40S ribosomal protein S13, putative | 8 |
| Tc00.1047053506113.60<br>Tc00.1047053507881.20 | Retrotransposon hot spot (RHS) protein, putative | 7 |
| Tc00.1047053506961.10 | Retrotransposon hot spot (RHS) protein, putative | 6 |
| Tc00.1047053504863.10 | Retrotransposon hot spot (RHS) protein, putative | 9 |
| Tc00.1047053508323.100 | I/6 autoantigen, putative | 10 |
| Tc00.1047053509693.80 | Hypoxanthine-guanine phosphoribosyltransferase, putative | 6 |
| Tc00.1047053504089.70<br>Tc00.1047053504001.20 | Hypothetical protein, conserved | 9 |
