# Supplementary material for: The Role of the Trypanosoma cruzi TcNRBD1 Protein in Translation
Source: PLoS One. 2016 Oct 19;11(10):e0164650. doi: 10.1371/journal.pone.0164650 (PMC5070865; doi:10.1371/journal.pone.0164650)
Supplement: S2 Table — (PDF) [file pone.0164650.s008.pdf]

S2 Table: Proteins identified by proteomic analysis in epimastigotes under nutritional stress.

ID	Description	Spectral count
Tc00.1047053503405.10	Glutamine synthetase, putative	7
Tc00.1047053509793.50	Hypothetical protein, conserved	7
Tc00.1047053509151.140	60S ribosomal protein L23a, putative	6
Tc00.1047053510767.20	60S ribosomal protein l36, putative	8
Tc00.1047053506977.49<br>Tc00.1047053509671.64	Ribosomal protein L36, putative	8
Tc00.1047053511211.160<br>Tc00.1047053511211.170<br>Tc00.1047053510439.61	Heat shock protein 70 (HSP70), putative	7
Tc00.1047053510089.110<br>Tc00.1047053510421.130	Hypothetical protein, conserved	7
Tc00.1047053510755.129<br>Tc00.1047053508413.44<br>Tc00.1047053511071.171<br>Tc00.1047053504037.30	60S ribosomal protein L12, putative	9
Tc00.1047053506297.160<br>Tc00.1047053506297.150<br>Tc00.1047053510101.170<br>Tc00.1047053510101.180	40S ribosomal protein S5, putative	8
Tc00.1047053508475.10<br>Tc00.1047053508823.120<br>Tc00.1047053508823.140	Ribosomal protein S20, putative	10
