# Supplementary material for: The Role of the Trypanosoma cruzi TcNRBD1 Protein in Translation
Source: PLoS One. 2016 Oct 19;11(10):e0164650. doi: 10.1371/journal.pone.0164650 (PMC5070865; doi:10.1371/journal.pone.0164650)
Supplement: S3 Table — (PDF) [file pone.0164650.s009.pdf]

S3 Table: mRNAs associated to TcNRBD1-mRNP in epimastigotes.

Feature ID	Product	Baggerley's test: 270 EPI vs Cont Weighted proportions log fold change	Baggerley's test: 270 EPI vs Cont - FDR p-value correction	Cont - Normalized means	270 EPI - Normalized means
TcCLB.510719.160	60S ribosomal protein L22, putative	1024,00	0,46%	0,00	216,17
TcCLB.507773.34	60S ribosomal protein L37, putative	1024,00	0,10%	0,00	92,29
TcCLB.506577.54	60S ribosomal protein L6, putative	1024,00	0,36%	0,00	59,21
TcCLB.504047.34	acylphosphatase, putative	1024,00	0,00%	0,00	88,13
TcCLB.504071.110	ama1 protein, putative	1024,00	0,00%	0,00	92,24
TcCLB.510801.10	calpain-like cysteine peptidase, putative	2,07	0,00%	26,40	111,18
TcCLB.506445.30	cation transporter, putative	2,94	0,72%	6,71	51,50
TcCLB.508173.60	cis-prenyltransferase, putative	1024,00	0,01%	0,00	62,93
TcCLB.503581.39	coatomer delta subunit, putative	1024,00	0,31%	0,00	65,67
TcCLB.510661.250	cystathione gamma lyase, putative	2,04	0,00%	33,46	138,04
TcCLB.511907.200	cysteine peptidase inhibitor, putative,chagasin, putative	2,65	0,00%	41,10	258,77
TcCLB.507949.4	cytidine triphosphate synthase, putative	1024,00	0,01%	0,00	78,30
TcCLB.503653.60	cytochrome b-domain protein, putative	2,07	0,67%	14,48	60,88
TcCLB.511025.110	dihydrolipoyl dehydrogenase, putative	2,96	0,00%	9,54	74,11

TcCLB.504175.40	dual specificity protein phosphatase, putative	2,09	0,01%	27,78	118,69
TcCLB.506679.70	eukaryotic translation initiation factor 6 (eIF-6), putative	3,02	0,00%	15,06	122,26
TcCLB.508827.30	eukaryotic translation initiation factor, putative	2,57	0,41%	19,92	118,57
TcCLB.508369.20	farnesyltransferase, putative,squalene synthase, putative	2,62	0,37%	11,26	69,48
TcCLB.508737.70	fatty acid desaturase, putative	2,52	0,00%	22,09	127,10
TcCLB.510669.20	Gim5A protein, putative,glycosomal membrane protein, putative	1024,00	0,00%	0,00	55,15
TcCLB.508479.110	glutathionylspermidine synthase, putative	3,51	0,00%	6,34	72,21
TcCLB.510283.70	glycosyl hydrolase-like protein, putative	2,29	0,12%	38,88	190,44
TcCLB.510819.60	heat shock protein HslVU, ATPase subunit HslU, putative	1024,00	0,36%	0,00	49,90
TcCLB.504071.40	hypothetical protein	1024,00	0,00%	0,00	92,39
TcCLB.506499.60	hypothetical protein	1024,00	0,00%	0,00	91,54
TcCLB.503411.10	hypothetical protein	1024,00	0,00%	0,00	86,03
TcCLB.506573.5	hypothetical protein	1024,00	0,00%	0,00	77,20
TcCLB.508099.40	hypothetical protein	1024,00	0,00%	0,00	64,07
TcCLB.510721.21	hypothetical protein	1024,00	0,12%	0,00	61,87
TcCLB.509521.10	hypothetical protein	1024,00	0,00%	0,00	57,88
TcCLB.511605.10	hypothetical protein	1024,00	0,00%	0,00	57,86
TcCLB.504081.370	hypothetical protein	1024,00	0,02%	0,00	55,13
TcCLB.504239.150	hypothetical protein	1024,00	0,43%	0,00	54,15
TcCLB.506821.10	hypothetical protein	1024,00	0,12%	0,00	53,76
TcCLB.506765.43	hypothetical protein	1024,00	0,36%	0,00	52,69
TcCLB.508355.270	hypothetical protein	1024,00	0,01%	0,00	52,40
TcCLB.511385.70	hypothetical protein	1024,00	0,00%	0,00	51,79

TcCLB.511815.90	hypothetical protein	2,94	0,69%	8,21	62,98
TcCLB.510479.30	hypothetical protein	2,88	0,00%	27,32	200,51
TcCLB.507517.50	hypothetical protein	2,86	0,07%	10,07	73,02
TcCLB.511223.10	hypothetical protein	2,80	0,42%	26,84	187,10
TcCLB.508243.36	hypothetical protein	2,73	0,00%	15,31	101,82
TcCLB.509039.40	hypothetical protein	2,56	0,00%	24,79	146,40
TcCLB.506993.190	hypothetical protein	2,32	0,02%	17,89	89,49
TcCLB.506705.50	hypothetical protein	2,30	0,00%	14,14	69,68
TcCLB.511593.65	hypothetical protein	2,13	0,09%	17,02	74,53
TcCLB.506529.541	hypothetical protein	2,11	0,00%	15,68	67,53
TcCLB.509295.90	hypothetical protein	2,09	0,00%	17,75	75,54
TcCLB.511771.75	hypothetical protein	2,07	0,76%	82,95	347,69
TcCLB.506989.110	hypothetical protein, conserved	1024,00	0,00%	0,00	159,68
TcCLB.506247.310	hypothetical protein, conserved	1024,00	0,00%	0,00	134,35
TcCLB.463451.4	hypothetical protein, conserved	1024,00	1,29%	0,00	117,38
TcCLB.511159.30	hypothetical protein, conserved	1024,00	0,00%	0,00	100,83
TcCLB.506869.30	hypothetical protein, conserved	1024,00	1,29%	0,00	85,20
TcCLB.503449.14	hypothetical protein, conserved	1024,00	0,00%	0,00	84,05
TcCLB.428403.5	hypothetical protein, conserved	1024,00	0,02%	0,00	76,50
TcCLB.510877.190	hypothetical protein, conserved	1024,00	0,12%	0,00	75,99
TcCLB.511181.150	hypothetical protein, conserved	1024,00	0,36%	0,00	73,87
TcCLB.509231.39	hypothetical protein, conserved	1024,00	0,12%	0,00	72,71
TcCLB.511393.49	hypothetical protein, conserved	1024,00	0,01%	0,00	71,20
TcCLB.509571.4	hypothetical protein, conserved	1024,00	0,00%	0,00	69,18

TcCLB.511509.21	hypothetical protein, conserved	1024,00	0,01%	0,00	67,88
TcCLB.509967.10	hypothetical protein, conserved	1024,00	0,00%	0,00	67,09
TcCLB.509161.120	hypothetical protein, conserved	1024,00	1,17%	0,00	66,86
TcCLB.508173.84	hypothetical protein, conserved	1024,00	0,00%	0,00	66,31
TcCLB.506221.70	hypothetical protein, conserved	1024,00	0,00%	0,00	62,77
TcCLB.504077.30	hypothetical protein, conserved	1024,00	0,00%	0,00	60,81
TcCLB.506265.110	hypothetical protein, conserved	1024,00	0,00%	0,00	60,76
TcCLB.508879.70	hypothetical protein, conserved	1024,00	0,01%	0,00	58,28
TcCLB.507765.90	hypothetical protein, conserved	1024,00	0,00%	0,00	58,21
TcCLB.506567.74	hypothetical protein, conserved	1024,00	0,10%	0,00	56,84
TcCLB.508209.159	hypothetical protein, conserved	1024,00	0,09%	0,00	55,42
TcCLB.506625.150	hypothetical protein, conserved	1024,00	0,36%	0,00	54,18
TcCLB.510359.220	hypothetical protein, conserved	1024,00	0,02%	0,00	53,09
TcCLB.507645.50	hypothetical protein, conserved	1024,00	0,00%	0,00	52,10
TcCLB.509601.100	hypothetical protein, conserved	1024,00	0,12%	0,00	51,42
TcCLB.511753.90	hypothetical protein, conserved	1024,00	0,12%	0,00	49,78
TcCLB.506237.19	hypothetical protein, conserved	1024,00	1,09%	0,00	49,71
TcCLB.507677.160	hypothetical protein, conserved	3,46	0,05%	7,53	82,99

TcCLB.508951.70	hypothetical protein, conserved	3,33	0,00%	5,32	53,44
TcCLB.508461.300	hypothetical protein, conserved	3,27	0,03%	7,62	73,71
TcCLB.506151.10	hypothetical protein, conserved	3,02	0,00%	6,15	49,92
TcCLB.506505.50	hypothetical protein, conserved	2,97	0,00%	6,41	50,11
TcCLB.511111.40	hypothetical protein, conserved	2,96	0,00%	7,89	61,29
TcCLB.511215.40	hypothetical protein, conserved	2,73	1,14%	8,81	58,53
TcCLB.509837.20	hypothetical protein, conserved	2,72	0,05%	9,18	60,52
TcCLB.504717.20	hypothetical protein, conserved	2,72	0,05%	7,89	52,04
TcCLB.506925.270	hypothetical protein, conserved	2,69	0,00%	13,97	90,15
TcCLB.510879.150	hypothetical protein, conserved	2,68	0,00%	20,19	129,20
TcCLB.509911.130	hypothetical protein, conserved	2,66	0,00%	12,35	78,27
TcCLB.508711.40	hypothetical protein, conserved	2,59	0,00%	9,33	56,23
TcCLB.509795.30	hypothetical protein, conserved	2,50	0,00%	9,71	55,06
TcCLB.451263.9	hypothetical protein, conserved	2,47	0,00%	16,10	89,36
TcCLB.507649.90	hypothetical protein, conserved	2,47	0,05%	28,16	155,77
TcCLB.510187.130	hypothetical protein, conserved	2,47	0,08%	9,35	51,71
TcCLB.507711.230	hypothetical protein, conserved	2,37	0,01%	10,61	54,95
TcCLB.511491.140	hypothetical protein, conserved	2,36	0,00%	20,64	106,32

TcCLB.506925.20	hypothetical protein, conserved	2,32	0,03%	13,66	68,32
TcCLB.509461.70	hypothetical protein, conserved	2,30	0,62%	10,14	49,99
TcCLB.506933.70	hypothetical protein, conserved	2,22	0,00%	21,88	101,85
TcCLB.507031.150	hypothetical protein, conserved	2,22	0,01%	12,69	58,95
TcCLB.508569.10	hypothetical protein, conserved	2,14	0,00%	15,28	67,35
TcCLB.508409.110	hypothetical protein, conserved	2,13	0,00%	34,69	151,99
TcCLB.510727.9	hypothetical protein, conserved	2,11	0,00%	23,06	99,55
TcCLB.507675.30	hypothetical protein, conserved	2,11	0,00%	19,09	82,23
TcCLB.511737.50	hypothetical protein, conserved	2,07	0,16%	100,43	421,44
TcCLB.511421.210	hypothetical protein, conserved	2,04	0,00%	24,93	102,84
TcCLB.508797.29	hypothetical protein, conserved	2,03	0,30%	15,30	62,40
TcCLB.507073.30	hypothetical protein, conserved	2,03	0,16%	18,52	75,53
TcCLB.510879.140	hypothetical protein, conserved	2,02	0,00%	48,28	196,00
TcCLB.504625.70	kinetoplast DNA-associated protein, putative	3,07	0,00%	8,16	68,52
TcCLB.511633.79	microtubule-associated protein, putative	2,69	0,00%	8,36	53,99
TcCLB.508461.284	mitochondrial carrier protein, putative	2,11	0,00%	12,30	52,97
TcCLB.504167.20	mucin TcMUCI, putative	1024,00	0,00%	0,00	90,65
TcCLB.508119.30	mucin TcMUCII, putative	1024,00	0,02%	0,00	50,61

TcCLB.511849.30	mucin-associated surface protein (MASP), putative	1024,00	0,01%	0,00	50,68
TcCLB.506965.140	mucin-associated surface protein (MASP), putative	2,09	0,00%	37,81	161,13
TcCLB.505843.40	NAD/FAD dependent dehydrogenase, putative	1024,00	0,00%	0,00	55,62
TcCLB.509567.40	nuclear transport factor 2, putative	1024,00	0,02%	0,00	78,95
TcCLB.509099.5	NUP-1 protein, putative	2,03	0,18%	64,87	264,54
TcCLB.505163.80	oligosaccharyl transferase subunit, putative	2,26	0,00%	11,42	54,88
TcCLB.509965.290	p22 protein precursor, putative	2,73	0,00%	20,19	134,26
TcCLB.511065.50	phosphatidylinositol 3-kinase, putative	1024,00	0,00%	0,00	75,27
TcCLB.511167.80	PIF1 helicase-like protein, putative,DNA repair and recombination protein, mitochondrial precursor, putative	2,47	0,08%	9,77	54,04
TcCLB.511483.40	prefoldin subunit 2, putative	1024,00	0,00%	0,00	51,05
TcCLB.511465.10	proteasome activator protein PA26, putative	2,19	0,04%	19,33	88,12
TcCLB.507775.50	proteasome alpha 7 subunit, putative	1024,00	1,09%	0,00	50,61
TcCLB.509287.20	protein kinase, putative	1024,00	0,11%	0,00	72,36
TcCLB.510247.10	R27-2 protein, putative	2,14	0,00%	14,27	62,72
TcCLB.511269.4	ras-related protein rab-5, putative	1024,00	0,00%	0,00	101,20
TcCLB.508647.40	replication factor C, subunit 1, putative	2,98	0,16%	7,83	61,58

TcCLB.509875.11	retrotransposon hot spot (RHS) protein, putative	1024,00	0,02%	0,00	88,45
TcCLB.508823.120	ribosomal protein S20, putative	1024,00	0,00%	0,00	64,46
TcCLB.509233.190	ribosomal protein S25, putative	2,37	0,00%	41,10	212,76
TcCLB.509317.60	RNA-binding protein, putative	1024,00	1,05%	0,00	91,26
TcCLB.507519.60	silent information regulator 2, putative,NAD-dependent SIR2, putative	1024,00	0,28%	0,00	50,76
TcCLB.511819.14	small nuclear ribonucleoprotein, putative	1024,00	0,00%	0,00	59,64
TcCLB.511111.10	translation initiation factor IF-2, putative	3,69	0,00%	5,46	70,74
TcCLB.510707.10	trans-sialidase, putative	1024,00	1,29%	0,00	194,82
TcCLB.511277.350	tubulin-specific chaperone, putative	1024,00	0,00%	0,00	97,94
TcCLB.504047.5	urocanate hydratase, putative	1024,00	0,10%	0,00	154,82
TcCLB.511511.3	zinc finger protein ZFP1	1024,00	0,00%	0,00	107,23
