# Supplementary material for: The Role of the Trypanosoma cruzi TcNRBD1 Protein in Translation
Source: PLoS One. 2016 Oct 19;11(10):e0164650. doi: 10.1371/journal.pone.0164650 (PMC5070865; doi:10.1371/journal.pone.0164650)
Supplement: S4 Table — (PDF) [file pone.0164650.s010.pdf]

S4 Table: mRNAs associated to TcNRBD1-mRNP in epimastigotes under nutritional stress.

Feature ID	Product	Baggerley's test: 270 STR vs Cont - Weighted proportions log fold change	Baggerley's test: 270 STR vs Cont - FDR p-value correction	Cont - Normalized means	270 STR - Normalized means
TcCLB.507681.160	40S ribosomal protein S24E, putative	1024,00	0,22%	0,00	179,11
TcCLB.508543.30	40S ribosomal protein S9, putative	2,25	1,47%	45,16	214,57
TcCLB.507053.10	50S ribosomal protein L7Ae, putative	1024,00	0,78%	0,00	58,93
TcCLB.508461.480	60S ribosomal protein L23, putative	2,17	0,10%	41,08	184,29
TcCLB.507601.70	dihydrolipoamide branched chain transacylase, putative	2,03	0,00%	16,45	67,26
TcCLB.506931.10	haloacid dehalogenase-like hydrolase, putative	2,06	0,36%	18,43	76,80
TcCLB.510439.61	heat shock 70 kDa protein, putative	2,10	0,71%	19,45	83,13
TcCLB.505071.40	hypothetical protein	2,68	0,00%	11,85	75,99
TcCLB.507225.19	hypothetical protein	3,44	0,00%	16,15	174,72
TcCLB.507237.30	hypothetical protein	1024,00	1,29%	0,00	62,25
TcCLB.507831.10	hypothetical protein	2,21	0,02%	12,39	57,24
TcCLB.508695.30	hypothetical protein	3,63	0,00%	7,94	98,10
TcCLB.509777.21	hypothetical protein	2,19	0,00%	576,34	2628,84
TcCLB.429229.10	hypothetical protein, conserved	2,02	1,04%	14,72	59,56
TcCLB.503527.20	hypothetical protein, conserved	2,07	0,00%	36,46	153,42
TcCLB.503887.40	hypothetical protein, conserved	2,21	0,02%	11,88	54,87
TcCLB.504097.4	hypothetical protein, conserved	2,06	0,03%	15,27	63,78
TcCLB.504741.40	hypothetical protein, conserved	3,21	0,67%	6,02	55,83
TcCLB.504839.60	hypothetical protein, conserved	2,31	0,22%	12,05	59,68
TcCLB.505807.160	hypothetical protein, conserved	2,07	0,41%	20,32	85,36
TcCLB.506155.110	hypothetical protein, conserved	4,05	0,00%	3,56	58,99
TcCLB.506227.220	hypothetical protein, conserved	2,28	0,00%	18,35	89,18
TcCLB.506247.40	hypothetical protein, conserved	2,25	0,63%	10,61	50,59

TcCLB.507927.60	hypothetical protein, conserved	3,18	0,00%	7,22	65,43
TcCLB.507993.30	hypothetical protein, conserved	1024,00	0,00%	0,00	65,88
TcCLB.508257.110	hypothetical protein, conserved	2,27	0,00%	21,78	104,86
TcCLB.508257.90	hypothetical protein, conserved	2,00	0,81%	17,70	71,00
TcCLB.508461.90	hypothetical protein, conserved	2,00	0,66%	32,13	128,91
TcCLB.508543.40	hypothetical protein, conserved	2,18	0,00%	16,06	72,73
TcCLB.508567.40	hypothetical protein, conserved	2,01	0,96%	24,62	99,17
TcCLB.508893.30	hypothetical protein, conserved	2,16	0,00%	13568,69	60464,31
TcCLB.509177.59	hypothetical protein, conserved	2,51	0,00%	38,86	221,23
TcCLB.509237.100	hypothetical protein, conserved	2,12	0,00%	20,25	88,13
TcCLB.509429.140	hypothetical protein, conserved	2,02	0,32%	13,63	55,14
TcCLB.509877.60	hypothetical protein, conserved	2,54	0,30%	15,97	93,15
TcCLB.510065.40	hypothetical protein, conserved	2,09	0,00%	16,99	72,50
TcCLB.510609.90	hypothetical protein, conserved	2,35	0,00%	10,84	55,10
TcCLB.511249.34	hypothetical protein, conserved	2,55	1,27%	8,90	52,23
TcCLB.511249.80	hypothetical protein, conserved	2,62	0,03%	120,25	741,74
TcCLB.511577.188	hypothetical protein, conserved	2,09	0,00%	23,96	102,18
TcCLB.511903.20	hypothetical protein, conserved	2,59	0,00%	12,50	75,32
TcCLB.408799.19	isopentenyl-diphosphate delta-isomerase, putative	1024,00	0,00%	0,00	63,80
TcCLB.510807.10	mucin TcMUCII, putative	2,14	0,00%	20,84	91,63
TcCLB.510289.6	nucleolar RNA-binding protein, putative	1024,00	0,01%	0,00	50,35
TcCLB.505999.20	phosphate-repressible phosphate permease, putative	2,25	0,00%	13,77	65,50
TcCLB.508461.80	prostaglandin F2alpha synthase	2,16	0,07%	147,56	660,46
TcCLB.507723.189	protein disulfide isomerase, putative	1024,00	0,65%	0,00	66,19
TcCLB.507251.20	ribosomal protein L21E (60S), putative	2,85	0,00%	35,94	259,25
TcCLB.509671.64	ribosomal protein L36, putative	2,65	0,00%	12,50	78,61
TcCLB.508119.144	ribosomal protein L38, putative	1024,00	0,00%	0,00	120,38
TcCLB.504105.94	ribosomal protein S25, putative	2,43	0,00%	25,91	139,51
TcCLB.506593.30	ribosomal protein S7, putative	2,36	0,02%	13,56	69,77
TcCLB.506625.70	RNA-binding protein, putative	2,51	0,00%	19,43	110,62
TcCLB.506155.50	vacuolar protein sorting-associated protein-like, putative	2,99	0,00%	6,72	53,46
